# Selective extracellular vesicle-mediated export of an overlapping set of microRNAs from multiple cell types

**DOI:** 10.1186/1471-2164-13-357

**Published:** 2012-08-01

**Authors:** Jasenka Guduric-Fuchs, Anna O’Connor, Bailey Camp, Christina L O'Neill, Reinhold J Medina, David A Simpson

**Affiliations:** 1 Centre for Vision and Vascular Science, Queen’s University Belfast, Belfast, Northern Ireland, UK

**Keywords:** microRNA, Deep sequencing, Extracellular vesicles, Exosomes

## Abstract

**Background:**

MicroRNAs (miRNAs) are a class of small RNA molecules that regulate expression of specific mRNA targets. They can be released from cells, often encapsulated within extracellular vesicles (EVs), and therefore have the potential to mediate intercellular communication. It has been suggested that certain miRNAs may be selectively exported, although the mechanism has yet to be identified. Manipulation of the miRNA content of EVs will be important for future therapeutic applications. We therefore wished to assess which endogenous miRNAs are enriched in EVs and how effectively an overexpressed miRNA would be exported.

**Results:**

Small RNA libraries from HEK293T cells and vesicles before or after transfection with a vector for miR-146a overexpression were analysed by deep sequencing. A subset of miRNAs was found to be enriched in EVs; pathway analysis of their predicted target genes suggests a potential role in regulation of endocytosis. RT-qPCR in additional cell types and analysis of publicly available data revealed that many of these miRNAs tend to be widely preferentially exported. Whilst overexpressed miR-146a was highly enriched both in transfected cells and their EVs, the cellular:EV ratios of endogenous miRNAs were not grossly altered. MiR-451 was consistently the most highly exported miRNA in many different cell types. Intriguingly, Argonaute2 (Ago2) is required for miR-451 maturation and knock out of Ago2 has been shown to decrease expression of other preferentially exported miRNAs (eg miR-150 and miR-142-3p).

**Conclusion:**

The global expression data provided by deep sequencing confirms that specific miRNAs are enriched in EVs released by HEK293T cells. Observation of similar patterns in a range of cell types suggests that a common mechanism for selective miRNA export may exist.

## Background

Membrane-bound extracellular vesicles (EVs), are referred to by various terms in the literature, including microvesicles or microparticles. However, they may be divided into two main types formed either by budding of the plasma membrane (shedding vesicles, ectosomes; approximately 100 nm −1 μm in diameter) or by exocytosis of multivesicular bodies (MVB) (exosomes; approximately 50–100 nm) [1-3]. While it is important to recognize that these two main populations of EVs have distinct biogenesis pathways, it remains technically challenging to isolate their respective pure populations due to their overlapping size distributions and most preparations are therefore heterogeneous [3]. EVs are targeted to specific recipient cells by surface receptors which mediate their internalisation and uptake of their content, comprising lipids, proteins, mRNAs and microRNAs (miRNAs) [2,4]. A significant physiological role for EVs is supported by their implication in cancer progression, angiogenesis, cell reprogramming and differentiation [5-9].

MiRNAs are a class of small RNA molecules that regulate gene expression by binding to specific mRNA targets and triggering their degradation or inhibiting their translation. Primary miRNAs (pri-miRNAs), transcribed either as independent genes or as introns of coding genes, are processed to form precursor hairpin loops (pre-miRNAs) and then mature miRNAs by the RNAse III enzymes Drosha and Dicer respectively. One strand of the mature miRNA is loaded into an RNA induced silencing complex (RISC), which mediates the interaction between the miRNA and its target mRNA molecules. Argonaute (Ago) proteins are part of the RISC and mediate cleavage of target mRNAs [10,11].

Recently, miRNAs have been found extracellularly, being encapsulated within vesicles (EVs), or associated with Ago2 or NPM1 proteins or high-density lipoproteins (HDL) [12-19]. EV preparations isolated by ultracentrifugation are heterogeneous and include both exosomes and other vesicles. Extracellular miRNAs have emerged as prognostic markers for various diseases, such as cancer and cardiovascular disorders [13,20-23]. Recent studies have demonstrated that miRNAs can be shuttled between cells in EVs and modulate gene expression [6,24-26] and control different physiological functions in the recipients [27-30].

It has been shown that miRNA export into exosomes occurs via the ceramide-dependent secretory pathway and independently of the endosomal sorting complex required for transport (ESCRT) [24]. Although the mechanism of release of shedding vesicles is less well known, the role of P4-ATPase and ESCRT has been recognized recently [31]. Several studies have suggested that the miRNA content of EVs does not simply reflect the miRNA repertoire of the cells of origin and that some miRNAs are selectively exported or retained within the cell [25,27,32-34]. However, the mechanism whereby specific miRNAs are selectively exported is unknown.

The potential for exploiting extracellular vesicles as vehicles for delivery of therapeutic molecules has been appreciated [35-38]. For example, EVs prepared from a patient’s own cells could potentially be enriched with specific siRNAs and/or miRNAs and used as a tool for therapeutic delivery of these regulatory RNA molecules. Alternatively, the therapeutic effect could be achieved by transplanting cells engineered to over-express critical miRNAs, which are delivered to the target cells via EVs[33,36]. To most effectively harness EVs as therapeutic tools, it will be crucial to understand and control how they are loaded with regulatory RNAs.

As a step towards understanding the mechanism of selective export of miRNAs to EVs we have applied deep sequencing to characterize the global expression pattern of small RNAs in HEK293T cells and the EVs that they release. Although the enrichment of overexpressed miRNA in EVs has been shown by RT-qPCR in HEK293T cells, mesenchymal stem cells, macrophages and immune cells [24,25,27,28,37], it is not known to what extent overexpressed miRNAs are exported into EVs and how they affect the export of endogenous miRNAs. To address these questions, small RNAs from cells transfected with miR-146a expressing plasmid and their EVs were also subjected to deep sequencing. Ectopic overexpression of miR-146a in HEK293T exosomes was previously shown to exert a functional effect upon recipient cells, including downregulation of target gene expression [24].

Comparison of our data with other publicly available data sets of miRNA expression in cells and their EVs revealed a subset of miRNAs that tend to be preferentially exported in various cell types, suggesting a common function in intercellular communication.

## Results

**Figure 1:**
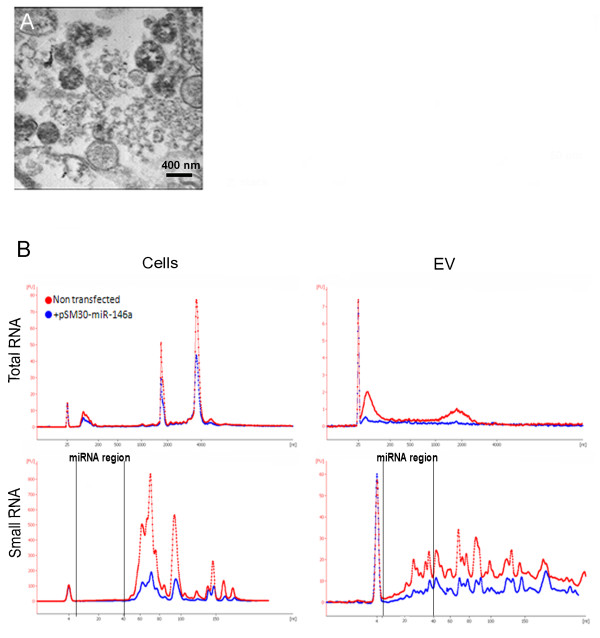
**Characterization of EVs and their RNA content.A**) Transmission electron microscopy of isolated EVs. **B**) Bioanalyzer profiles of the total and small RNAs of the HEK293T cells and EVs. (Lower peaks for the transfected cells and EVs (blue) are due to less RNA loaded).

EVs from HEK293T cells were obtained by ultracentifugation of conditioned media at 100000 *g*. The presence of spherical membrane bound structures of variable diameter up to approximately 1000 nm in these preparations was confirmed by transmission electron microscopy (Figure 1A). RNA extracted from cells and EVs was analyzed on a Bioanalyzer (Agilent Technologies). The shape of the RNA profiles observed when comparing RNA from pSM30-miR-146a transfected cells and EVs and their non-transfected counterparts were similar (Figure 1B). Total RNA from EVs was enriched in small RNAs and lacked ribosomal RNA peaks typical for cell RNA profiles (although a low broad peak was observed at the level of 18 S RNA). Analysis of the small RNA profiles of EV samples revealed enrichment of sequences of the size expected for miRNAs, with a peak at approximately 20 nt.

To compare the global miRNA profile of non-transfected and pSM30-miR-146a transfected cells and their EVs, small RNA libraries were prepared and sequenced on an Illumina Genome Analyzer. Raw reads were subjected to adaptor removal and aligned to miRBase (Release 17, http://www.mirbase.org/) [39]. Both raw and processed data has been deposited in Gene Expression Omnibus (GEO: http://www.ncbi.nlm.nih.gov/geo/), with accession number GSE38916. The total numbers of reads >15 bp matching each miRNA were calculated. These values were then normalized to number of reads per million mapped (RPMM) in each library, to enable direct comparison of expression levels. As a measure of the relative levels of expression of individual miRNAs in cells and EVs, the log (base two) of the ratio of the read number in EVs over that in cells was calculated (log_2_ (EV/cell)). The miRNA expression data for cells and EVs is presented in Additional file 1: Table S1.

**Figure 2:**
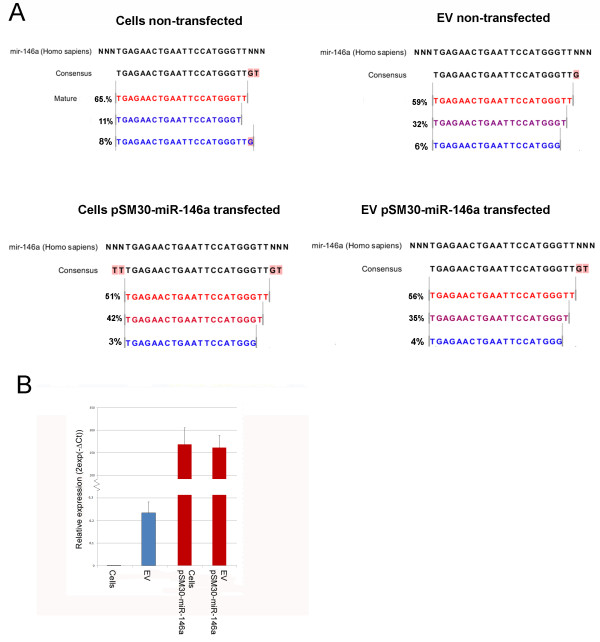
**Over-expression of miR-146a.A**) Deep sequencing reads mapped to the miR-146a pre-miR sequence. The most frequent isomiR (red) of miR-146a expressed exogenously following transfection with pSM30-146a (transfected cells and EVs) is represented by a similar percentage of reads as for endogenous miR-146a. **B**) RT-qPCR quantification of miR-146a in non-transfected and pSM30-146a transfected HEK293T cells.

The enrichment of miR-146a in cells and EVs following transfection with pSM30-miR-146a was striking: Mir-146a was enriched from 10 RPMM (0.1% of all reads) to 317,863 RPMM (32%) in cells and from 1,321 RPMM (1.3%) to 440,496 RPMM (44%) in EVs. Sequencing revealed that overexpressed miR-146a was correctly processed, with the most prevalent isomiR being the same in all cell and EV libraries (Figure 2A) and represented by approximately half of the reads. RT-qPCR from independent pSM30-miR-146a transfections confirmed consistently high overexpression of miR-146a in both cells and EVs (Figure 2B).

**Figure 3:**
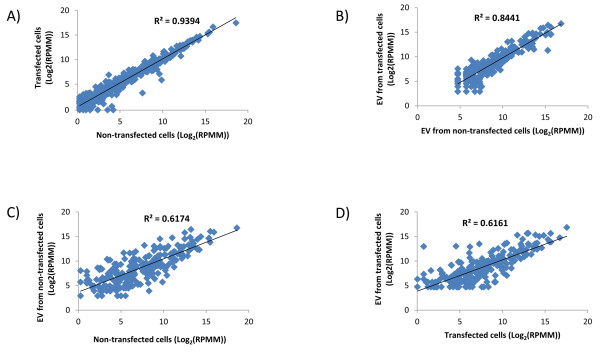
**MiRNA expression in cells and EVs.** Scatter plots of the miRNA expression levels in cells and EVs of non-transfected and pSM30-miR-146a transfected cells. **A**) There was extremely high correlation between miRNA expression values in non-transfected and transfected cells (R^2^ = 0.9). **B**) Strong correlation was also observed between EVs from non-transfected and transfected cells (R^2^ = 0.8). **C**) Correlation between transfected and non-transfected cells and **D**) between EVs from transfected and non-transfected cells was much lower (R^2^ = 0.6). For pSM30-miR-146a transfected cells and EVs expression levels were renormalized to RPMM following exclusion of miR-146a reads.

To evaluate how the overexpression of miR-146a affected the proportions of other, endogenous miRNAs, we excluded reads for miR-146a from transfected cells and EVs and re-normalized the expression values of all other miRNAs to reads per million mapped (RPMM). This enabled direct comparison between read numbers in transfected and non-transfected cells and EVs. This re-normalisation revealed extremely high correlation between miRNA expression values in non-transfected and transfected cells (R^2^ = 0.9) (Figure 3A). Moreover, high correlation was also observed between EVs from non-transfected and transfected cells (R^2^ = 0.8) (Figure 3B). However, correlation between both transfected and non-transfected cells and their respective EVs was much lower (R^2^ = 0.6) (Figure 3C,D). From all miRNAs that had been detected in both non-transfected cells and EVs, only 19.5% had log_2_ (EV/cell) between −1 and +1, therefore being represented by similar number of reads in cells and EVs.

**Figure 4:**
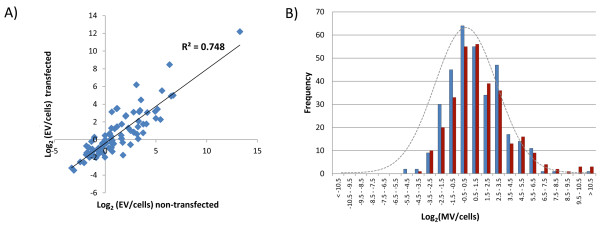
**Relative expression between cells and microvesicles.A**) The ratios of the expression levels of individual miRNAs between cells and EVs remained reasonably constant following overexpression of exogenous miR146a expression, as illustrated by the strong correlation between the values. **B**) When expression levels in both cells and EVs were normalized to RPMM, more miRNAs were preferentially released in EVs than retained in cells: The histograms for both non-transfected (blue) and miR-146a overexpressing samples (red) are shifted to the right relative to a normal distribution (grey line).

The miRNAs preferentially retained within the cell or released in EVs are highly consistent even after over-expression; a scatter plot of log_2_ ratios of miRNAs represented by more than 1000 reads either in cells or EVs revealed relatively high correlation (R^2^ = 0.748)(Figure 4A). The most retained and most released miRNAs which are represented by more than 1000 reads either in cells or in EVs are listed in Tables 1 and 2 respectively. MiR-218 was found to be the most retained and miR-451 the most exported miRNA. If miRNAs are incorporated passively into EVs as a random sample of the cellular population one would expect the ratios of EV:cell expression values to be approximately 1:1 when both values are normalized to million reads mapped (i.e. a distribution of log_2_ (EV/cell) centred on zero). However, we observed a skewed distribution (Figure 4B), with more miRNAs represented by higher RPMM values in EVs than cells (i.e. with a positive log_2_ (EV/cell) ratio). The values of the positive ratios representing the most released miRNAs, were more extreme than the most negative values. However, several very highly expressed miRNAs had negative values (i.e. preferentially retained within the cell).

**Table 1:** miRNAs most preferentially released into EVs

**miRNA**	**Non-transfected cells & EVs**			**miR-146a - Transfected cells & EVs**		
	**RPMM cells**	**RPMM EV**	**Log**_**2**_**(EV/Cells)**	**RPMM cells**	**RPMM EV**	**Log**_**2**_**(EV/Cells)**
**mir-451**	0.4	3945.0	**13.3**	1.7	7982.5	**12.2**
**mir-150**	0.4	2475.0	**12.6**	0.3	663.0	**10.9**
mir-146a*	10.4	1321.2	**7.0**	317863.3*	440496.3*	**0.5**
**mir-320c**	24.3	2347.5	**6.6**	57.8	1803.4	**5.0**
**mir-143**	39.9	3232.5	**6.3**	8327.3	354.4	**8.5**
**mir-720**	65.4	3157.5	**5.6**	3208.9	46.2	**5.5**
**mir-125a-5p**	214.7	9360.0	**5.4**	439.0	2121.6	**2.3**
**mir-486**	105.7	3900.0	**5.2**	72.8	7637.8	**6.7**
**mir-149**	89.0	3142.5	**5.1**	85.3	928.2	**3.4**
**mir-2110**	43.5	1380.0	**5.0**	175.0	928.2	**2.4**
**mir-320a**	762.1	23190.0	**4.9**	1250.0	11483.2	**3.2**
**mir-197**	472.4	8347.5	**4.1**	724.0	6046.6	**3.1**
**mir-339**	91.4	1515.0	**4.1**	178.0	610.0	**1.8**
**mir-99b**	2065.2	27420.0	**3.7**	4900.0	8353.8	**0.8**
**mir-4286**	108.5	1245.0	**3.5**	72.1	1617.7	**4.5**
**mir-92b**	515.1	5542.5	**3.4**	484.0	4800.1	**3.3**
**mir-222**	774.1	7882.5	**3.3**	1200.0	10634.5	**3.1**
**mir-423-5p**	5737.2	55762.5	**3.3**	5290.0	22435.9	**2.1**
**mir-877**	467.2	4425.0	**3.2**	2000.0	2121.6	**0.1**
**mir-191**	9501.8	88717.5	**3.2**	15000.0	40893.8	**1.4**
**mir-1292**	140.0	1222.5	**3.1**	256.0	397.8	**0.6**
**mir-1246**	782.8	6570.0	**3.1**	156.0	11456.6	**6.2**

Those miRNAs represented by at least 1000 reads in either cells or EV are listed. * For the transfected cells and EVs the RPMM values were calculated after removal of the reads from over-expressed miR-146a, to facilitate comparison with non-transfected samples (the RPMM values shown for miR-146a are those prior to this normalisation).

**Table 2:** miRNAs most preferentially retained within cells

**miRNA**	**Non-transfected cells & EVs**			**miR-146a - Transfected cells & EVs**		
	**RPMM cells**	**RPMM EV**	**Log**_**2**_**(EV/Cells)**	**RPMM cells**	**RPMM EV**	**Log**_**2**_**(EV/Cells)**
**mir-218**	1342.24	135.00	**−3.31**	1460.00	132.60	**−3.46**
**mir-15a**	2411.56	435.00	**−2.47**	2120.00	371.28	**−2.51**
**mir-140**	1181.04	277.50	**−2.09**	1130.00	185.64	**−2.61**
**mir-148b**	18756.99	5047.50	**−1.89**	17300.00	5383.56	**−1.68**
**mir-378**	397404.00	109560.00	**−1.86**	192000.00	117695.76	**−0.71**
**mir-16**	7225.49	2100.00	**−1.78**	7190.00	2625.48	**−1.45**
**mir-20a**	45087.00	14460.00	**−1.64**	52700.00	14957.28	**−1.82**
**mir-424**	1230.92	397.50	**−1.63**	878.00	477.36	**−0.88**
**mir-18a**	4535.03	1477.50	**−1.62**	4520.00	1405.56	**−1.69**
**let-7f**	16253.27	5520.00	**−1.56**	31964.69	11055.00	**−1.53**
**let-7 g**	4999.47	1950.00	**−1.36**	5580.00	4826.64	**−0.21**
**mir-130b**	2194.90	877.50	**−1.32**	2180.00	769.08	**−1.50**
**mir-15b**	2820.93	1147.50	**−1.30**	2780.00	1087.32	**−1.35**
**mir-598**	1066.13	435.00	**−1.29**	1400.00	397.80	**−1.82**
**mir-26b**	1594.80	660.00	**−1.27**	1440.00	689.52	**−1.06**
**mir-340**	4503.91	2137.50	**−1.08**	1870.00	2280.72	**0.29**

Those miRNAs represented by at least 1000 reads in either cells or EV are listed. * For the transfected cells and EVs the RPMM values were calculated after removal of the reads from over-expressed miR-146a, to facilitate comparison with non-transfected samples.

**Figure 5:**
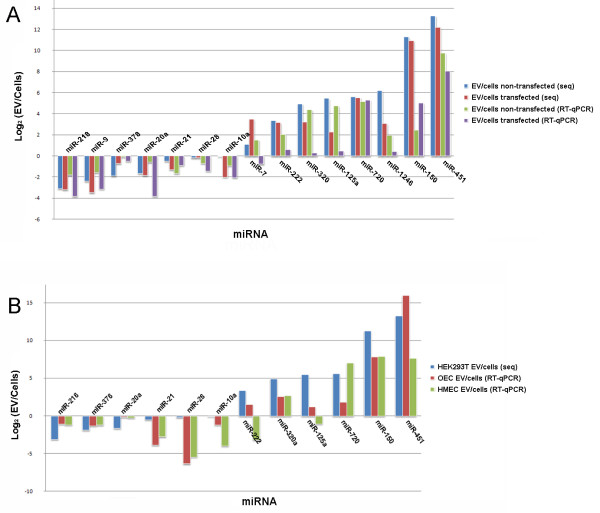
**Comparison of EV:cells ratios obtained by RT-qPCR and deep sequencing.A**) Confirmation of EV:cells ratios by RT-qPCR for miRNA in biological replicates of non-transfected and pSM30-146a transfected HEK293T cells. **B**) Comparison of EV:cells ratios from HEK293T cells (deep sequencing) with HMEC-1 and OEC cells (RT-qPCR). RT-qPCR data were normalized to miR-26a.

To confirm sequencing data, we performed RT-qPCR on RNA samples isolated from biological replicates of non-transfected and pSM30miR-146a transfected cells and their EVs. MiR-26a was chosen for normalization of the RT-qPCR data because this miRNA had relatively high expression (~2000 RPMM) and the numbers of reads in cells and EVs were similar (EV:Cell ratio ~1). The miRNA EV:cell ratios obtained by PCR correlated with sequencing data and confirmed retained and exported miRNAs (Figure 5A).

The most highly exported miRNA in EVs in our data, miR-451, was also reported previously to be highly exported from breast cancer cells lines and mesenchymal stem cells [25,33]. This was intriguing, and it prompted us to speculate that the same miRNAs may be preferentially exported or retained in various cell types. We therefore tested two other cell types, namely a human microvascular endothelial cell line, (HMEC-1) and primary outgrowth endothelial progenitor cells (OECs) with the same panel of primers used to confirm sequencing data in HEK293T cells. For the 12 miRNAs expressed in both endothelial cells and EVs the proportions retained or released followed a similar pattern to that observed in HEK293T cells (Figure 5B). Two miRNAs, miR-1246 and miR-9 were not detected and miR-7 was not detected in endothelial EVs.

**Figure 6:**
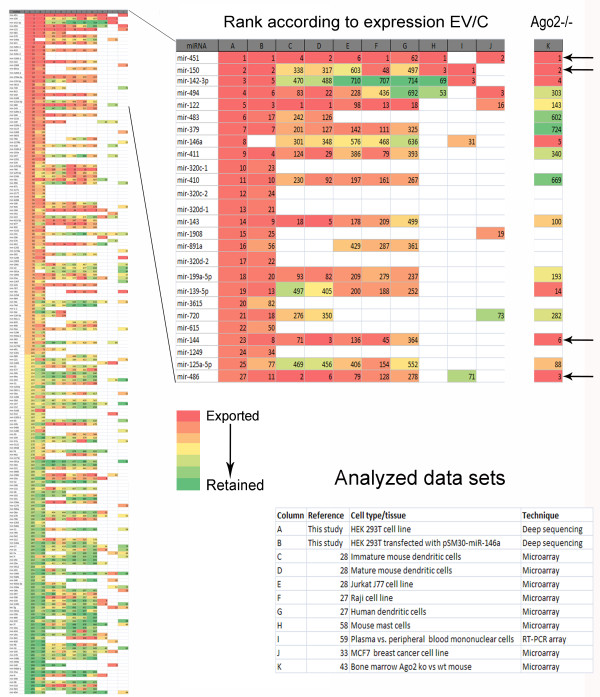
**Overlap between miRNAs exported by different cell types.** All miRNAs identified in HEK293T cells were coloured according to their EV:cells ratio with most preferentially exported red and most retained within the cell green. The 100 miRNAs with the smallest and largest log_2_(EV/cell) values are shown on the left. Publicly available data sets for various cell types were analysed in a similar fashion and compared with our data. The pattern of exported and retained miRNAs shows similarities between the different data sets. The most exported miRNAs cluster predominantly in the upper part of the graph which is enlarged in the central panel to show the identities of the consistently exported miRNAs. The right-hand column (column K) illustrates the miRNAs ranked according to reduction in Ago2 knockout tissue relative to wildtype reported by Yang *et al.*[43]. Arrows indicate miRNAs with high ranks in both Ago2 and other data sets. Blank white spaces indicate that a miRNA was either not detected or not assayed.

To obtain a broader view of miRNA export in various cells types, we retrieved publicly available data (microarray and RT-qPCR arrays) on miRNA expression in various cell types and their EVs (microvesicles, exosomes). The ratios (or delta Ct for RT-qPCR data) between the expression levels in EVs and cells were calculated and the miRNAs ranked accordingly. Comparison of the ranks of the miRNAs present in our data with their ranks in the other datasets revealed correlations between the different cell types. This was visualized by assigning a color for each miRNA according to its rank in a particular data set: Figure 6A shows the ranks of the top and bottom 100 miRNAs sorted according to the data from HEK293T non-transfected cells (all miRNAs are listed in Additional file 2: Table S2). MiR-451, the most highly enriched miRNA in HEK293T EVs, was among the top 6 exported miRNAs in 6 out of 8 of the other datasets.

MiR-451 is the only reported miRNA that is not processed by Dicer, but instead uses an Ago2-mediated pathway for its maturation [40-42]. It is tempting to speculate that this unique processing pathway might be connected with the high extracellular export of miR-451. In this case one might expect the most exported miRNAs to also be the most affected by loss of Ago2 activity. We therefore ranked miRNAs according to the reduction in their expression in bone marrow of Ago2 ko mice compared to the wild type [43]. Remarkably, in addition to miR-451, several other miRNAs among the top 6 affected by the Ago2 deletion (miR150, miR-486, and miR-144) were also found to be highly exported in several data sets (see column (K) in Figure 6).

**Figure 7:**
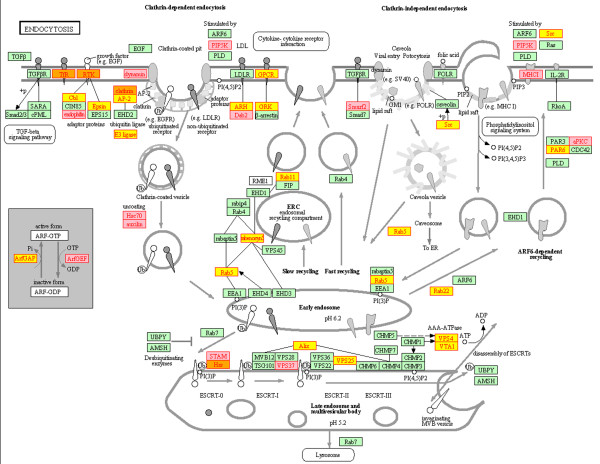
**Targets of selectively exported miRNAs are involved in endocytosis.** The endocytosis pathway (Kegg: hsa:04144) is depicted with the target genes of the 10 most exported miRNAs highlighted. Those genes predicted by Diana microT-CDS are highlighted in yellow, those by Starbase (supported by CLIP-Seq data) in pink and those by both approaches in orange.

As an initial step towards understanding the function of the preferentially exported miRNAs we attempted to determine the genes which are being targeted by these miRNAs. It is difficult to predict these interactions with certainty, but the algorithms available are improving. We employed microT-CDS, which has recently been shown to perform well in terms of sensitivity and specificity compared to other available algorithms [44]. The 2760 genes predicted to be targeted by one or more of the ten most selectively exported miRNAs are listed in Additional file 3: Table S3. Functional analysis of these genes revealed that the most enriched pathway was endocytosis (Kegg hsa04144) (Benjamini-corrected p-value = 0.05) which contained 43 predicted target genes (Figure 7). All the enriched pathways and gene ontologies are listed in Additional file 4: Table S4 as well as clusters of functionally related terms, which include transcriptional regulation, intracellular transport and organelles. Recently developed techniques based upon crosslinking of RISC proteins with their target mRNAs have facilitated experimental determination of miRNA:target interactions [45,46]. Therefore, to complement the *in silico* target prediction, the genes with target sites for the same 10 most exported miRNAs predicted by any algorithm that are supported experimentally by CLIP-Seq reads available in Starbase [47] were downloaded (Additional file 3: Table S3). These genes were also significantly enriched in the endocytosis pathway (Benjamini-corrected p-value = 0.0009) and because only three of the predicted target genes in this pathway are common (Figure 7) the *in silico* and experimental predictions provide largely independent associations with endocytosis. From the total of 43 microT-CDS and 39 Starbase predicted target genes 7 were common.

## Discussion

MiRNAs are involved in regulation of a wide array of biological processes, from development to immunity. Extracellular and circulating miRNAs have been found both in the non-vesicular fraction and encapsulated in EVs. They have been shown to associate with lipids (HDL) and proteins (Ago2 and NPM1). While the role of EV miRNAs in cell-to-cell communication has now been widely accepted [3] and HDL-mediated miRNA transfer has been shown to regulate recipient cell gene expression [19], there is still no direct evidence that miRNAs associated with proteins act in a paracrine fashion [16-18].

Realisation of the potential importance of miRNAs in cell-to-cell communication has triggered research into the mechanisms of miRNA release and their effects upon recipient cells [4]. Evidence is accumulating that miRNA export into EVs is selective, but it is not known how this export is regulated. Several studies have compared the miRNA content of cells and EVs using microarrays or RT-qPCR. Deep sequencing is an alternative approach for measuring miRNA expression which has the advantage of providing a digital measure that does not require an internal standard for normalisation. This is particularly important when comparing miRNA levels between cells and EVs, where no common internal standards have been identified. Although several previous studies have applied deep sequencing in similar scenarios, they involved fewer reads and the expression of only selected miRNAs was reported [48,49]. Here we report a global comparison of the miRNA pools in HEK293T cells and their EVs. Our analysis confirmed previous observations that some miRNAs are preferentially exported and found to be enriched in EVs.

Enrichment of a miRNA in EVs after its overexpression in cells has been measured previously by RT-qPCR. However, it is important to reveal how such overexpression compares with and affects expression and export of endogenous miRNAs. We found overexpressed miR-146a to contribute almost a third of all reads in cells and even more in EVs, representing almost half of their cargo. It is very important for the miRNA therapeutic field to understand the consequence of miRNA overexpression. Our data suggest that if a miRNA is overexpressed in a specific cell type the possibility that a significant amount of this miRNA will be exported and potentially affect other cells should be considered. Although we cannot assess the effect of miR-146a overexpression upon the absolute expression of other miRNAs, this effect is likely to be similar in both cells and EVs. However, our data demonstrates that the relative expression levels of endogenous miRNAs (and their EV:cell miRNA ratios) remain very stable and are largely unaffected by the miRNA overexpression. The fact that selective miRNA export is tightly regulated suggests that it plays an important role in cell physiology. This will be an important consideration not only for further studies into the mechanism of miRNA export but also for the development of methods for enrichment of EVs with miRNA for therapies. It is possible that the overexpressed miRNA and endogenous miRNAs use different export routes and therefore do not affect each other. Since our EV preparation does not distinguish between exosomes and shedding vesicles we cannot exclude that one of these is preferentially used to carry overexpressed miRNA. Fractionation of EVs will be necessary to further examine this possibility. Nevertheless, there may be an overlap between the two pathways, as a recent study has suggested that shedding vesicles and exosomes require the same GTPase (RAB-11) and ESCRT proteins for their biogenesis and therefore may be regulated by the same mechanisms, regardless of their distinct cellular localization [31].

Comparison of our data with publicly available expression sets from other cell types revealed that some miRNAs tend to be universally exported. It is possible that this reflects release of the same type of vesicles (eg exosome) by all the cell types analysed. The commonly exported miRNAs possibly fulfill a common function in intercellular communication. MiR-451 was found to be the miRNA with the highest ratio between expression levels in EVs and cells. In relation to its maturation miR-451 is a non-canonical miRNA, processed by the Ago2 protein, in contrast to other miRNAs that commonly require Dicer for this process [40,41,43]. Ago2 has been shown previously to be associated with both EV and non-vesicular miRNAs [17,18,49]. Comparison of miRNA expression between cells and their EV counterparts from other published data sets reveals miR-451 to be one of the most highly exported miRNAs in multiple cell types. Deletion of Ago2 in bone marrow leads to reduced expression of several other miRNAs in addition to miR-451 [43], notably some of the miRNAs that are most preferentially exported from various different cell types. These data suggest that there may be a connection between Ago2 processing and the selection of miRNAs for export. Recent studies have demonstrated the physical colocalisation of RISC and MVBs and their functional interactions in regulating miRNA loading and RNA silencing [49,50]. Being a key component of RISC, Ago2 may potentially be involved in directing miRNA towards exosomal secretion. However it has not been investigated whether Ago2 mediated miR-451 processing also occurs within RISC and in colocalisation with MVB membrane, or in another subcellular compartment. Future studies are required to characterize miRNA export into specific EV populations and must also address the challenge of dissecting the function of Ago2 in miRNA processing from its potential role in miRNA export.

## Conclusions

Specific endogenous miRNAs, most notably miR-451 are selectively released from HEK293T cells in EVs by a process that may involve Ago2. Conservation amongst the miRNAs exported by different cells suggests that this process may be widely used to regulate similar target cell functions. Enrichment in genes involved in endocytosis amongst the targets of the most selectively exported miRNAs suggests that these functions may include intercellular vesicular transport itself. The demonstration that EV miRNA content can be manipulated by cellular overexpression of a specific miRNA bodes well for future therapeutic use of this system to deliver miRNAs/siRNAs to target cells.

## Methods

### Cell culture

HEK293T cells from the American Type Culture Collection (ATCC) were cultured in DMEM (Life technologies, Paisley, UK) supplemented with 10% fetal calf serum (FCS) and 100 μg/ml Primocin™ (InvivoGen). Human microvascular endothelial cells (HMEC-1) [51] were cultured in MCDB 131 medium (Life technologies) containing 10% FCS, L-glutamine (2 mM), epidermal growth factor (10 ng/ml, Life technologies), hydrocortisone (1 μg/ml, Life technologies), and 100 μg/ml Primocin. As described previously [52], OECs were obtained from the mononuclear cell fraction of umbilical cord blood, obtained under full ethical approval from The Office for Research Ethics Committees Northern Ireland (ORECNI). Cells were resuspended in EGM-2 medium (Lonza) supplemented with 10% FCS and seeded on 24 well culture plates pre-coated with rat tail collagen type 1 (BD Biosciences) at a density of 1×10^7^/ml All cells were cultured under standard conditions at 5% CO_2_, 37°C, and 95% humidity.

### MiRNA cloning

The mature miRNA sequences for miR-146a, were inserted into pSM30 vector according to the published protocol [53]. Briefly, the oligos containing the miR-146a mature sequence and the miR-30 loop sequence were designed as follows: top strand miR-146a oligo: 5'- AGCGGACCCATGGAATTCAGTTCTCATAGTGAAGCCACAGATGTATGAGAACTGAATTCCATGGGTT-3' and the bottom strand miR-146a oligo: 5'-GGCAAACCCATGGAATTCAGTTCTCATACATCTGTGGCTTCACTATGAGAACTGAATTCCATGGGTC-3'). Oligos were synthesized and HPLC purified by Eurogentec (Seraing, Belgium). Five μl of each oligo (100 μM stock solution) were mixed with 8 μl water and 2 μl of 10 x restriction buffer 3 (New England Biolabs), heated at 95°C for 4 minutes and left at room temperature for 15 minutes to anneal. Annealed oligos were diluted 2500 x to get 10 nM for ligation. Annealed oligos were ligated into pSM30 vector using Rapid Ligation Kit (Roche) with 15:1 insert:vector ratio. pSM30 empty vector was digested with BSMBI restriction enzyme and linearized plasmid was gel purified. DH5α competent cells were transformed with the ligation reaction and positive colonies were selected on agar plates supplemented with kanamycin.

### Transfection

HEK293T cells were seeded at 3×10^4^ cells/ml into T75 flasks and transfected after 24 hours with Turbofect in vitro transfection reagent (Fermentas, York, UK) according to the manufacturer’s instructions. After 16 hours media were replaced with media containing 10% serum depleted of EVs by centrifugation for 2 hours at 100000 *g*. EVs were collected from the conditioned media after 48 h hours.

### Isolation and characterisation of EVs

EVs were isolated as described previously [25,54]. Cell debris was removed by centrifugation at 2000 *g* for 30 minutes and vesicles were isolated by ultracentrifugation at 100000 *g* for 1 hour.

For Electron microscopy HEK293T EVs pellets were fixed in 2.5% glutaraldehyde in 0.1 M sodium cacodylate buffer at 4°C overnight. After post-fixation in 1% osmium tetroxide, pellets were dehydrated with increasing alcohol concentrations and embedded in Spurr resin. Ultrathin sections were prepared using an ultramicrotome, placed on copper grids (Agar Scientific Ltd, UK), stained with uranyl acetate and lead citrate, and examined using a JEOL 100 CX transmission electron microscope.

### RNA extraction

RNA was extracted using a miRNeasy kit (Qiagen, Crawley, UK) or High pure miRNA isolation kit (Roche, Burgess Hill, UK) according to the manufacturers’ protocols. RNA purity and quantity was determined using a Nanodrop spectrophotometer (Thermo Scientific) or Qubit fluorimeter (Life Technologies). RNA integrity was assessed using RNA 2000 and small RNA chips on a Bioanalyzer (Agilent).

### Deep sequencing

Small RNA libraries were prepared using a sample prep kit v1.5 kit (Illumina) following the manufacturer’s protocol. Cluster generation and sequencing on a Genome Analyzer II was performed at the Trinity Genome Sequencing Laboratory, Dublin (http://www.medicine.tcd.ie/sequencing). Sequencing data were analyzed using Genomics workbench software (CLCbio, Aarhus, Denmark). After removal of adapter sequences reads >15 bp were aligned to miRBase (Release 17) for annotation, allowing no more than 2 mismatches. Reads were normalized to reads per million mapped (RPMM) to enable comparison between different libraries. If a miRNA was found in both EV data sets but was detected in only one data set from cells (due to its low expression value) it was assigned one read in the other cell data set for the purpose of calculating an EV:cells ratio.

Target genes were predicted for miRNAs of interest using the microT-CDS algorithm available at http://www.microrna.gr/microT-CDS with a strict miTG Score Threshold of 0.8 [44]. Lists were integrated using Microsoft Access and pathway analysis of the KEGG database[55] performed using the The Database for Annotation, Visualization and Integrated Discovery (DAVID) v6.7[56] available at http://david.abcc.ncifcrf.gov/. Predicted target sites supported by CLIP-Seq reads were downloaded from Starbase (http://starbase.sysu.edu.cn/) [47].

### RT-qPCR

Specific miRNAs were amplified using a modified version of the method described by Shi and Chiang [57]. One microgram of RNA was polyadenylated using poly(A) polymerase (PAP; Ambion, Foster City, CA) at 37°C for 1 hour in a 25 μl reaction mixture. RNA was then reverse transcribed with 200 U of SuperScript III reverse transcriptase (Life technologies, Paisley, UK) and 0.5 μg poly (T) adapter. Primers for specific miRNAs were designed from miRNA sequences obtained from miRBase and our sequencing data and synthesized by Eurogentec (Seraing, Liège, Belgium) (Additional file 5: Table S5). The reverse primer was the 3' adapter primer (3' RACE outer primer in the FirstChoice RLM-RACE kit; Ambion).

RT-qPCR was performed using Maxima SYBR Green qPCR mastermix (Fermentas) in 10 μl reactions containing 2 μl of 1:15 cDNA dilution. Reactions were performed on a LightCycler 480 (Roche), with the initial preincubation at 50°C for 2 min and activation at 95°C for 10 min, followed by 40 cycles at 95°C for 15 s, and 60°C for 60 s, with fluorescence acquired after 15 s of the 60°C step. Primer efficiencies were determined using serial dilutions of pooled cDNA and the reactions were optimized to achieve efficiencies of ~2 for all primers used. The RNA from three independent experiments was analyzed and all PCR reactions were performed in triplicates. Gene expression data were normalized to miR-26a. The relative expression was determined as 2^−ΔCt^, where ΔCt = Ct(miRNA)-Ct(miR-26a).

### Analysis of publicly available miRNA expression data

Publicly available data were included in analysis if they had expression data for EVs (exosomes, microvesicles) and corresponding cells. Microarray data were downloaded from GEO database [27,28,33] or retrieved from supplementary data [58,59]. Difference between expression in cells and EVs was determined as the ratio of expression EV:cells for microarrays or as delta Ct for RT-qPCR arrays and miRNAs were ranked accordingly. Using Microsoft Access miRNAs that are present in our data were pulled out from all the other data sets with their own ranks kept. Colours were assigned to miRNAs according to their ranks using Microsoft Excel (conditional formatting).

## Abbreviations

CLIP-Seq: Crosslinking immunoprecipitation sequencing; EVs: Extracellular vesicles; HMEC-1: Human microvascular endothelial cell line; MVB: Multivesicular bodies; OECs: Outgrowth endothelial progenitor cells; RISC: RNA-Induced Silencing Complex; RPMM: Reads per million mapped.

## Competing interests

The authors declare that they have no competing interests.

## Authors’ contributions

JG-F contributed to the design of the study, carried out library preparation, sample preparation and RT-qPCR, participated in the analysis of sequence data and drafted the manuscript. AOC constructed the miR-146a expression vector. BC performed RT-qPCR expression analyses. CLO isolated OECs. RJM isolated OECs and assisted with manuscript preparation. DAS conceived, designed and co-ordinated the study, analysed the sequence data and drafted the manuscript. All authors read and approved the final manuscript.

## Supplementary Material

Additional file 1 **Table S1.** RPMM cells and EVs. Click here for file

Additional file 2 **Table S2.** Full list of ranked miRNAs shown in Figure 6. Click here for file

Additional file 3 **Table S3.** Target predictions. Click here for file

Additional file 4 **Table S4.** Functional annotation. Click here for file

Additional file 5 **Table S5.** Primer sequences. Click here for file
